# Synthesis of Block Copolymer Brush by RAFT and Click Chemistry and Its Self-Assembly as a Thin Film

**DOI:** 10.3390/molecules25204774

**Published:** 2020-10-17

**Authors:** Hajeeth Thankappan, Mona Semsarilar, Suming Li, Yung Chang, Denis Bouyer, Damien Quemener

**Affiliations:** 1 Institut Européen des Membranes, IEM-UMR 5635, Univ Montpellier, ENSCM, CNRS, 34095 Montpellier, France; ajithmphil@gmail.com (H.T.); mona.semsarilar@umontpellier.fr (M.S.); Suming.Li@umontpellier.fr (S.L.); Denis.Bouyer@umontpellier.fr (D.B.); 2 Department of Chemical Engineering, R&D Center for Membrane Technology, Chung Yuan Christian University, 200, Chung-Bei Rd., Chungli, Taoyuan, 320, Taiwan; changyung0307@gmail.com

**Keywords:** block copolymer brush, click chemistry, self-assembly, thin film

## Abstract

A well-defined block copolymer brush poly(glycidyl methacrylate)-*graft*-(poly(methyl methacrylate)-*block*- poly(oligo(ethylene glycol) methyl ether methacrylate)) (PGMA-*g*-(PMMA-*b*-POEGMA)) is synthesized via grafting from an approach based on a combination of click chemistry and reversible addition-fragmentation chain transfer (RAFT) polymerization. The resulting block copolymer brushes were characterized by ^1^H-NMR and size exclusion chromatography (SEC). The self-assembly of the block copolymer brush was then investigated under selective solvent conditions in three systems: THF/water, THF/CH_3_OH, and DMSO/CHCl_3_. PGMA-*g*-(PMMA-*b*-POEGMA) was found to self-assemble into spherical micelle structures as analyzed by transmission electron microscopy (TEM) and dynamic light scattering (DLS). The average size of the particles was much smaller in THF/CH_3_OH and DMSO/CHCl_3_ as compared with the THF/water system. Thin film of block copolymer brushes with tunable surface properties was then prepared by the spin-coating technique. The thickness of the thin film was confirmed by scanning electron microscopy (SEM). Atom force microscopy (AFM) analysis revealed a spherical morphology when the block copolymer brush was treated with poor solvents for the backbone and hydrophobic side chains. The contact angle measurements were used to confirm the surface rearrangements of the block copolymer brushes.

## 1. Introduction

In recent years, special attention has been paid to understanding the macromolecular architecture–property relationship thanks to the occurrence of a large polymer diversity. Particularly true for copolymers, modern synthesis techniques enable to target different compositions and topologies such as linear, cyclic, star, graft, network, and hyperbranched [1]. Block copolymer brushes are one example of an interesting variant combining topology and composition with recent works exploring potential applications in colloid stabilization, tailoring surface properties, and “chemical gates” [2,3,4]. Their tunable size and shape-persistence also make block copolymer brushes well suited for the in vivo delivery of therapeutic agents [5]. The three well known strategies of grafting-through, grafting-onto, and grafting-from [6,7,8,9,10] are commonly used to prepare well-defined block copolymer brushes with controlled molecular weight and narrow molecular weight distributions. Controlled radical polymerization (CRP) techniques [11], including reversible addition-fragmentation chain transfer polymerization (RAFT) [12], atom transfer radical polymerization (ATRP) [13], and nitroxide-mediated radical polymerization (NMP) [14], were used with success in the synthesis of functional (co)polymers with complex macromolecular architectures. RAFT polymerization in particular is a powerful approach for the macromolecular synthesis of a broad range of well-defined polymers [15,16]. The versatility of the method is proven by its compatibility with a very wide range of functional monomers and reaction conditions. allowing for example to go to the completion of monomer conversion without observing an adverse effect on the macromolecular structure and molecular weight distribution [17]. To further increase the architectural possibilities, polymer chain conjugation through highly efficient chemical reactions, known as click chemistry, has been reported [18,19,20]. For example, few works have described the use of copper(I)-catalyzed azide-alkyne cycloaddition (CuAAC) in combination with RAFT polymerization to synthesize brush block copolymer architecture [21,22,23]. The azide and alkyne-functionality are better introduced in the R group of the RAFT agent, so it could be preserved a posteriori at one chain end, while possibly removing the RAFT agent at the other end. As an alternative approach, direct polymerization of an alkyne or azide monomer gives a polymer that can be post-functionalized by reacting with the corresponding component [24]. However, to synthesize highly complex block copolymers, architecture still remains a challenge and only very few robust protocols for efficient synthesis of such polymers have been reported owing to their inherent difficult synthesis. Indeed, the synthesis of brush type polymer having complex macromolecular composition and topolgy is still rare in the literature.

For block copolymer brushes, we could simply refer to a tight assembly of block copolymer chains, connected by one end to an interface [25,26]. However, in this work, we only consider block copolymer chains attached together by one end to a polymer backbone. When an amphiphilic block copolymer side chain forms the brushes, self-assembly into micelles could occur under selective solvent conditions [21,27,28,29]. Indeed, the self-assembly of a block copolymer brush may constitute a new class of advanced thin-film materials that can be used in microelectronic, optical, and optoelectronic devices. The conceptual ability to tune morphology of a given block copolymer brush by a simple selective solvent choice is of great interest and will facilitate further advances in nanotechnology applications.

The underlying idea of this work is to synthesize block copolymer brushes by the combination of RAFT and click chemistry approaches. Here, the block copolymer brush is made from poly(glycidyl methacrylate)-*graft*-(poly(methyl methacrylate)-*block*-poly(oligo(ethylene glycol) methyl ether methacrylate)) (PGMA-*g*-(PMMA-*b*-POEGMA)). With this aim, multifunctional azido homopolymer was prepared by RAFT polymerization of PGMA, the introduction of azide groups resulting from the subsequent ring opening of the oxirane rings. On the other hand, unprotected alkyne end functional RAFT agent was prepared and used in the polymerization of PMMA and POEGMA to form alkyne terminated POEGMA-*b*-PMMA block copolymer. Finally, the azide and alkyne functionalised polymers were attached together via CuAAC reactions. Solutions of the resulting block copolymer brush under selective solvent conditions were studied by dynamic light scattering and transmission electron microscopy, whereas solid thin films, prepared by spin coating, were characterized by scanning electron microscopy and atomic force microscopy.

## 2. Results

### 2.1. Synthesis of Alkyne Terminated RAFT Chain Transfer Agent (CPADB-Alk)

**Scheme 1:**
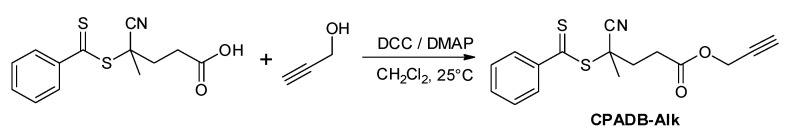
Synthesis of 4-cyano-4(phenylcarbonothioylthio) pentanoic acid (CPADB)-Alk.

Synthesis of CPADB-Alk, as shown in Scheme 1, involves coupling of propargyl alcohol and 4 cyano-4-(phenylcarbonothioylthio)pentanoic acid via Steglich esterification [30] using *N*,*N’*-dicyclohexylcarbodiimide (DCC) as coupling agent with catalytic amount of 4-dimethylaminopyridine, in dry dichloromethane for 20 h. *N*,*N’*-dicyclohexylcarbodiimide and the RAFT ended carboxylic acid are able to form an *O*-acylisourea intermediate. The addition of *N*,*N’*-dicyclohexylcarbodiimide and 4-dimethylaminopyridine, in dichloromethane solution, was carried out slowly to avoid the formation of N-acyl impurity.

**Figure 1:**
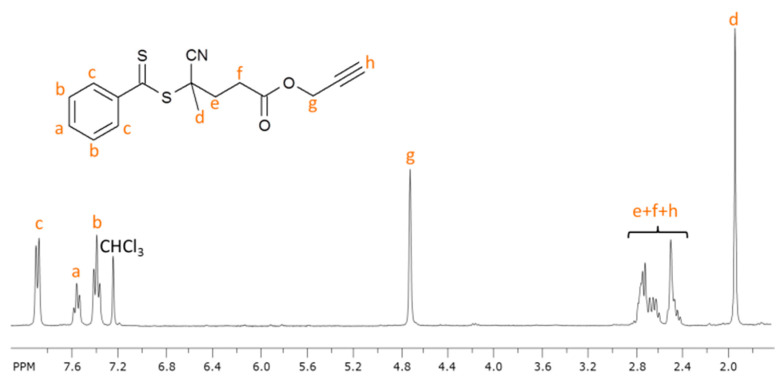
^1^H-NMR spectrum of 4-cyano-4(phenylcarbonothioylthio) pentanoic acid (CPADB)-Alk.

The propargyl alcohol was then added slowly to the activated carboxylic acid to form the stable dicyclohexylurea. The ^1^H-NMR spectrum and peak assignments of CPADB-Alk are presented in Figure 1. The peak at 4.7 ppm (d,-C(O)-O-C*H_2_*) indicates in particular the success of the reaction.

### 2.2. Synthesis of Poly(oligo(ethylene glycol) methyl ether methacrylate)-b-poly(methyl methacrylate) (POEGMA-b-PMMA-Alk) Block Copolymer

**Scheme 2:**
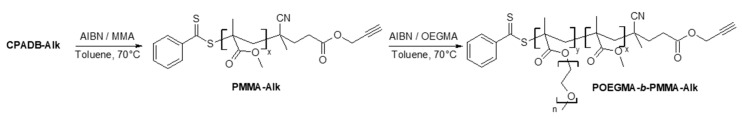
Synthesis of poly(oligo(ethylene glycol) methyl ether methacrylate)-*b*-poly(methyl methacrylate) (POEGMA-*b*-PMMA)-Alk block copolymer. AIBN, azobisisobutyronitrile; MMA, methyl methacrylate.

**Figure 2:**
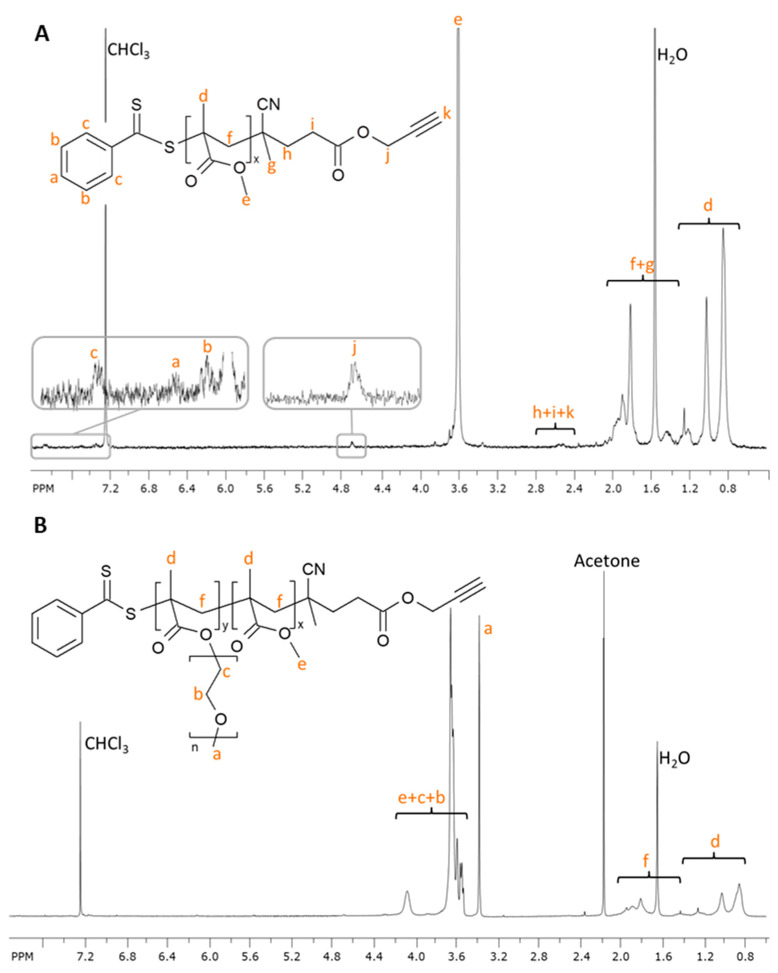
^1^H-NMR spectra in CDCl_3_ of (**A**) PMMA-Alk and (**B**) POEGMA-*b*-PMMA-Alk block copolymer.

Scheme 2 shows the synthetic route for the preparation of POEGMA-*b*-PMMA-Alk block copolymer. At the first step, the alkyne terminated PMMA macro-CTA is synthesized in toluene at 70 °C using CPADB-Alk. The polymer structure was confirmed by ^1^H-NMR (Figure 2A) and the molar mass was characterized by SEC (e.g., M_n,SEC_ = 1800 g·mol^−1^, Ð = 1.09).

The RAFT polymerization of OEGMA was then performed at 70 °C using PMMA-Alk and AIBN in toluene. The obtained POEGMA-*b*-PMMA-Alk block copolymer was then characterized by ^1^H-NMR (Figure 2B) and SEC (e.g., M_n,SEC_ = 3100 g·mol^−1^, Ð = 1.13). The molecular characteristics of all alkyne terminated PMMA and POEGMA-*b*-PMMA-Alk block copolymers determined by SEC are summarized in Table 1.

### 2.3. Synthesis of Poly(glycidyl methacrylate)(PGMA) and Poly(glycidyl methacrylate azide)(P(GMA-N_3_))

**Scheme 3:**
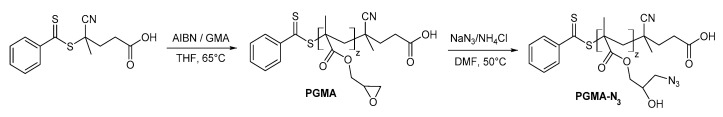
Synthesis of PGMA and P(GMA-N_3_). GMA, glycidyl methacrylate.

The RAFT polymerization of GMA has been already reported for homopolymers [31], but not with CPADB as a RAFT agent. The synthesis of PGMA with CPADB was carried out in toluene at 70 °C, but the polymerization medium quickly precipitates within 1 h as a result of the extremely low solubility of PGMA growing chains in toluene, resulting in a poor control of the polymerizations (Ð ~ 1.5, data not shown). The RAFT polymerization of GMA (Scheme 3) was then initiated in THF for 2 h at 70 °C. The SEC results suggest a good control over the molar mass and dispersity (e.g., M_n,SEC_ = 3300 g·mol^−1^, Ð = 1.12). A ring-opening reaction was then performed on PGMA homopolymers (Scheme 3) by reaction with NaN_3_ and NH_4_Cl in order to functionalize each monomer unit with an azido group [32]. The presence of NH_4_Cl could quench the alkoxide anion formed during the ring opening reaction. The molecular characteristics of all PGMA and azide terminated PGMA determined by SEC are summarized in Table 1. It should be noted that the molecular weights of the azide-modified polymer measured by SEC are higher than unmodified PGMA precursor, in agreement with the works of Yang et al. and Li et al. [33,34,35]. Here, the molecular weight increase as measured by SEC (Table 1) is slightly larger than expected and is accompanied by an increase of the dispersity. As no crosslinking can occur in this previously reported reaction, it is believed that the appearance of polar groups such as OH and N_3_ could drive aggregation and/or interaction with the SEC columns.

**Figure 3:**
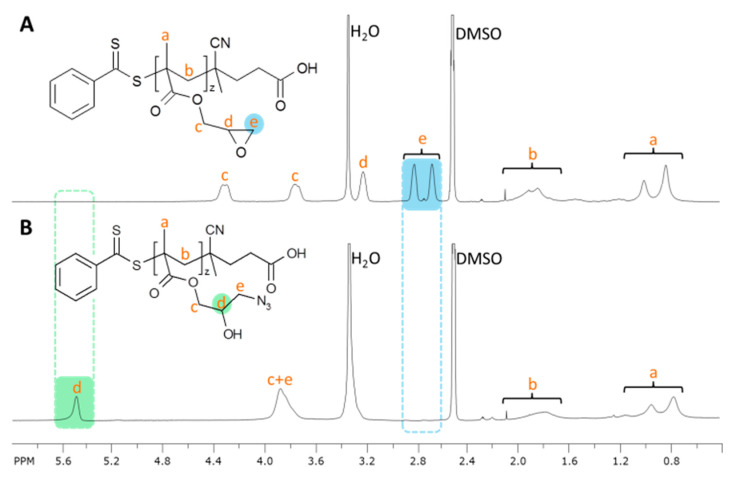
^1^H-NMR spectra in DMSO-*d*_6_ of (**A**) PGMA and (**B**) P(GMA-N_3_).

The ^1^H-NMR spectra of PGMA before and after azide reaction (Figure 3A,B), however, confirmed the efficient functionalization: the signals corresponding to the epoxide groups (protons d and e) disappeared, while new corresponding signals were found at 5.51 and 3.88 ppm, which was ascribed to the proton of CH-O, CH_2_-O, and CH_2_-N_3_, evidencing a quantitative ring opening of the epoxide groups. The results indicate that the azide anion attacked preferentially at the less substituted carbon atom of the epoxide ring. This result is consistent with previous studies reported by Matyjaszewski and Lim group [32,36]. An estimation of M_n_ by ^1^H-NMR could not be done because a complete loss of aromatic end groups at 7–7.9 ppm was also observed (Figure S1). Indeed, a cleavage of dithiobenzoate end groups in the presence of azide occurred [37], which was confirmed by a change of the color of the reaction medium from pink to white.

### 2.4. Block Copolymer Brush from Click Reaction between P(GMA-N_3_) and POEGMA-b-PMMA-Alk

**Scheme 4:**
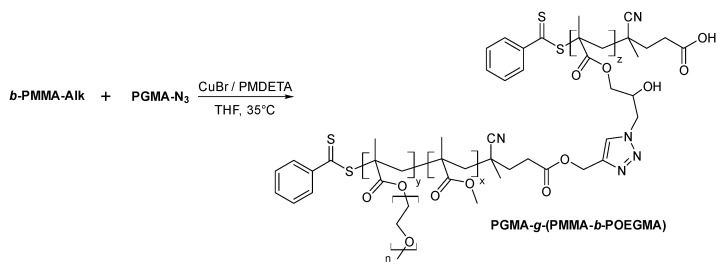
Synthesis of block copolymer brush (PGMA-*g*-(PMMA-*b*-POEGMA)).

**Figure 4:**
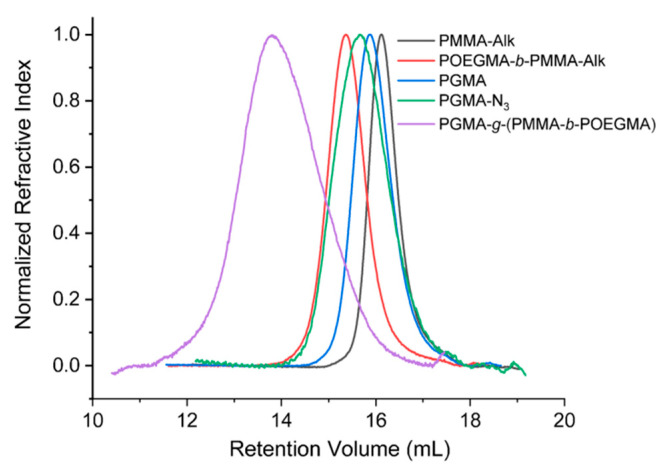
Molecular weight distribution of the block copolymer brush and all its precursors.

Copper catalyzed cycloadditions of azide–alkyne to triazoles coupling reactions are highly efficient and have been successfully used in novel polymer materials’ synthesis. The click reaction between alkyne terminated POEGMA-*b*-PMMA-Alk block copolymer (M_n,SEC_ = 3100 g·mol^−1^, Ð = 1.13) and P(GMA-N_3_) (M_n,SEC_ = 15,800 g·mol^−1^, Ð = 1.15) was carried out with CuBr as the catalyst (Scheme 4), PMDETA as the ligand, and THF as the solvent. Firstly, azide (1.0 mol eq.) and alkyne (1.0 mol eq.) fragments were dissolved in THF in the presence of PMDETA (2.5 mol eq. w.r.t. azide fragment) at 25 °C. CuBr (2.5 eqv w.r.t. azide fragment) was then added. The reaction progress was monitored after 6 and 16 h. The SEC analyses (data not shown) using THF eluent show molecular weight distributions with shoulder even after 16 h, indicating an incomplete reaction between azide and alkyne components. Additional reactions were thus performed in which the polymeric alkyne to azide ratio was increased twofold, and the catalyst, CuBr/PMDETA, was used at 5.0 eqv relative to azide groups. The SEC results (Figure 4) after 20 h show a single peak without an appreciable shoulder. The catalyst was removed from the polymer solution by passing through a neutral alumina column, washed with ammonia solution, followed by subjecting the product to dialysis (MW Cutoff 12k). The resulting solution was freeze dried and then dissolved in THF and precipitated in diethyl ether.

**Figure 5:**
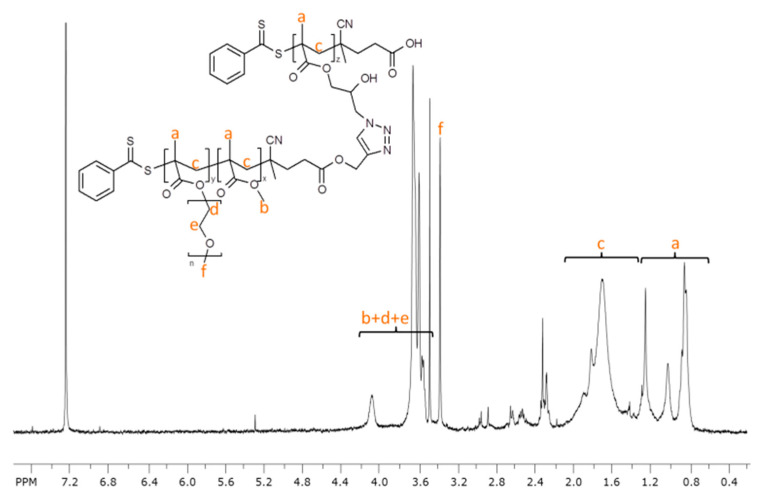
^1^H-NMR spectrum in CDCl_3_ of PGMA-*g*-(PMMA-*b*-POEGMA) block copolymer brush.

The block copolymer brush was characterized by ^1^H-NMR and SEC. ^1^H-NMR (Figure 5) confirms the presence of the azido functionalized PGMA and POEGMA-*b*-PMMA block copolymer groups. The SEC traces given in Figure 4 clearly show a shift in the molecular weight distribution in the case of block copolymer brush with respect to the azido and alkyne functionalized polymers (M_n,SEC_ = 86,000 g·mol^−1^). Although the molecular weight of the brush polymers is greater than that of its precursor, the increase in M_n_ is not perfectly matched with the expected value. As explained by Lian et al. and Li et al. [21,38], this could be due to the compact grafting structure and the high segment density of the brush polymers. It should be noted here that the calculation of M_n_ by ^1^H-NMR was not reliable because of an overlap of the peaks of the backbone and side chains.

The width of the molecular weight distribution is acceptable, although it has increased to Ð ≈ 1.3 as compared with the corresponding precursors. Detailed information of the block copolymer brushes with varying backbone and side chains is presented in Table 1. The block copolymer brush 1 (Table 1) PGMA_23_-*g*-(PMMA_18_-*b*-POEGMA_4_) was chosen as a good compromise in terms of block length with a relatively short PMMA, enabling enough mobility during the film formation.

**Figure 6:**
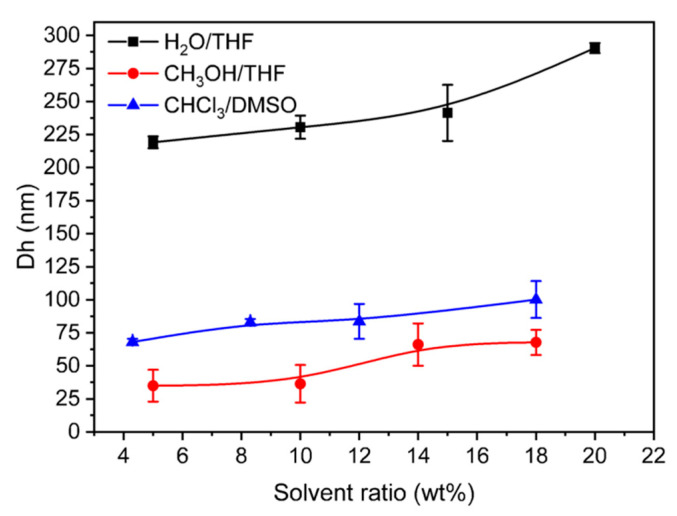
Hydrodynamic diameter of the block copolymer brush (sample 1) under selective solvent conditions as measured by dynamic light scattering (DLS). The solvent ratio indicates the proportion of the selective solvent (H_2_O, CH_3_OH, and CHCl_3_).

Three different solvent systems were explored: THF/water, THF/methanol, and DMSO/CHCl_3_. The copolymer was first dissolved in a non-selective solvent (THF or DMSO), at a concentration of 1 mg·mL^−1^. A selective solvent (water, methanol or CHCl_3_) was then slowly added to the mixture under vigorous stirring until reaching the desired solvent ratio. The solution was let to stir for an additional 10 min before being analyzed by DLS (Figure 6).

**Figure 7:**
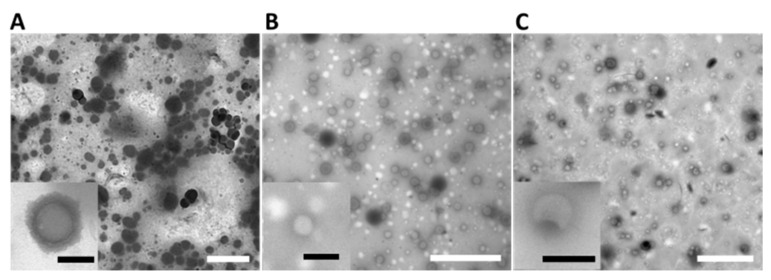
Transmission electron microscopy (TEM) pictures of block copolymer brush particles (sample 1) in selective solvent systems. (**A**) THF/water (90/10 *w*/*w*). (**B**) THF/CH_3_OH (82/18 *w*/*w*). (**C**) DMSO/CHCl_3_ (82/18 *w*/*w*). Scale bars are all of 1 µm for the main pictures and of 125 nm for the enlargements.

Considering the THF/water system, the hydrodynamic size remained stable around 230 nm up to a water concentration of about 10 wt%. However, above this value, a slight increase of the size was observed and visible aggregates were formed in the solution. The morphologies of the block copolymer brush that had formed in water (10 wt%) were verified by TEM (Figure 7A). The size of the particles in THF/water mixture (230 nm) agrees with size from TEM results in dry state (194 nm), although the polydispersity seems higher. Here, it should be noted that water is a poor solvent for PMMA and P(GMA-N_3_), but a good solvent for POEGMA. Thus, PMMA and P(GMA-N_3_) would collapse in contact with water, while the soluble POEGMA chains in the solvents stay solvated and help to stabilize the particles. A core shell spherical morphology was observed accordingly in TEM. Mo et al. in 2017 have also reported a spherical morphology from ternary graft copolymer in water. The morphology was explained by a decreasing solubility of P(GMA-N_3_) in H_2_O, which makes the skeleton chain more compact and the contraction of the hydrophobic side chains results in a reduction of steric repulsion between the two grafts, thus facilitating the continuation of the dorsal chain collapse in water [39]. Li et al. and Szymusiak et al. have also reported spherical morphology from graft copolymers having a PGMA backbone and POEGMA side chains [34,40]. However, Lian et al. have instead reported a vesicular morphology with PGMA-*g*-(PEO/PS-*b*-PNIPAM) macromolecular brush in water. This may be because of the lower critical solution temperature of PNIPAM probably altering the morphology in aqueous solution [21]. Here, only a relatively small change in the particle size was found despite the large variations of the structure as reported in Table 1.

For example, the hydrodynamic diameter of PGMA_23_-*g*-(PMMA_18_-*b*-POEGMA_4_) (sample 1, Table 1) was found to increase only from 219 nm to 248 nm with PGMA_31_-*g*-(PMMA_33_-*b*-POEGMA_4_) (sample 2, Table 1) and to 231 nm with PGMA_23_-*g*-(PMMA_33_-*b*-POEGMA_9_) (sample 4, Table 1). Replacing water by methanol as a selective solvent has decreased the hydrodynamic diameter of the particles from 219 to 34 nm with a solvent ratio THF/CH_3_OH of 95/5 (*w*/*w*). Like water, methanol is a poor solvent for PMMA block, however, it is a partially poor solvent for P(GMA-N_3_) and a good solvent for POEGMA. Thus, PMMA, localized between the PGMA backbone and the POEGMA side graft, would entirely collapse, while the main backbone would stay slightly solvated and the POEGMA will again stay fully solvated. The DLS size slightly increases when the proportion of methanol increases. It should be noted here that the morphology of graft copolymer observed by Mo et al. was different from our current results. A worm-like structure was observed in their case rather than spherical morphology for PGMA-*g*-(PCEMA-*r*-PtBA-*r*-MPEG) in methanol. Methanol was found to be a poor solvent for P(GMA-N_3_) and PCEMA, but a good solvent for the PtBA and MPEG side chains. Strong repulsive interactions between the soluble side chains of PtBA and MPEG were suspected to prevent the formation of spherical micelles [39]. As mentioned before, methanol is a good solvent only for POEGMA, the terminal block on side chains in this work. When its concentration is increased, an extension of the block will increase the hydrodynamic micelle size, as demonstrated in Figure 6. In a similar trend as with the THF/water system, a change of the brush copolymer structure will not considerably alter the particle size. For example, the hydrodynamic diameter slightly increases from 34 nm for PGMA_23_-*g*-(PMMA_18_-*b*-POEGMA_4_) to 54 nm for PGMA_31_-*g*-(PMMA_33_-*b*-POEGMA_4_) (5/95 *w*/*w* CH_3_0H/THF). The last solvent system tested was DMSO/CHCl_3_ with DMSO as a good solvent for all the polymer fragments and chloroform good for the block copolymer side chains, but poor for the PGMA backbone. The low solubility of the PGMA backbone containing triazole rings in chlorinated solvent such as dichloromethane was already reported in the literature [39]. A similar initial decrease of the particle size was observed from 219 nm to 69 nm (compared with THF/water mixture) with a solvent ratio DMSO/CHCl_3_ of 95/5 *w*/*w*. As described in the literature, this change in size is most probably due to a change in the aggregation number, because block copolymer brushes could form unimolecular structures, showing better stability in concentration thanks to high intramolecular interactions [28,39]. The morphologies of the block copolymer brushes that had formed in DMSO/CHCl_3_ (18 wt%) and THF/methanol (18 wt%) were verified by TEM (Figure 7B,C). The size of the particles obtained from DLS in DMSO/CHCl_3_ mixture (100 nm) and THF/methanol (67 nm) agrees with the size from TEM results in dried state, at 93 nm and 75 nm, respectively. The above results clearly demonstrated that the size of the particles was highly influenced by the solvent mixture.

### 2.5. Thin Films’ Preparation

**Figure 8:**
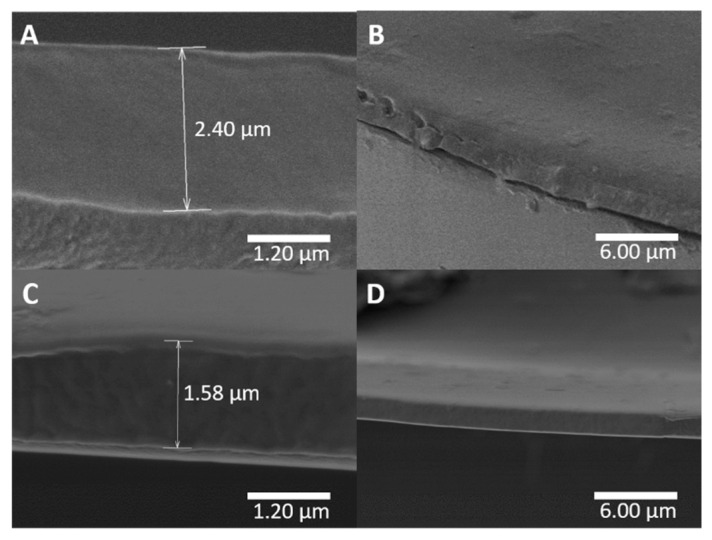
Scanning electron microscopy (SEM) pictures (cross section and top view) of the block copolymer brush films made from sample 1. (**A**) and (**B**) Film prepared from THF polymer solution. (**C)** and (**D**) Film prepared from the THF/water solvent system.

For the particles being dispersed in solution, a step of polymer concentration is thus required to produce the expected solid films. However, in the solvent systems DMSO/CHCl_3_ and THF/methanol, CHCl_3_ and THF are highly volatile, which results in a progressive change of the solvent composition over the course of the evaporation. Therefore, the aggregate morphology is changed in the final materials. We have thus focused on the THF/water system exclusively. Two types of films were prepared, where either pure THF or THF/water solutions of PGMA_23_-*g*-(PMMA_18_-*b*-POEGMA_4_) (sample 1) block copolymer brush (15 wt%) were spin-coated onto silicon wafers. The films were then characterized by SEM after drying under nitrogen (Figure 8).

**Figure 9:**
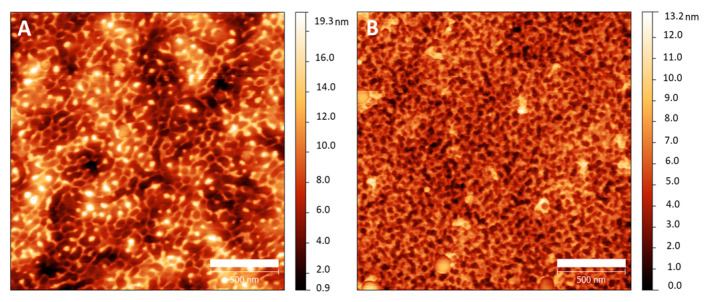
Atom force microscopy (AFM) pictures of block copolymer brush thin films prepared from pure THF (**A**) or THF/water (**B**) solution of sample 1. Scale bars: 500 nm.

Both films present a smooth surface and a symmetrical morphology (homogeneous cross section), as expected from the spin-coating technique. The thickness was measured to 2.4 µm from the pure THF solution and 1.6 µm from the THF/water system, the difference being explained by a difference of the initial solution viscosity. Additionally, AFM was used to characterize the thin film of block copolymer brushes. Figure 9A,B show the AFM images of thin film prepared from THF and THF/water mixture.

**Figure 10:**
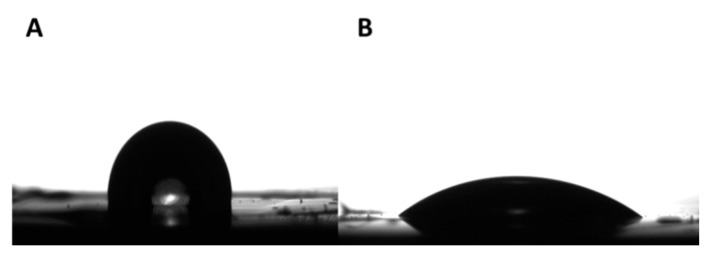
Water contact angle of block copolymer brush thin films prepared from pure THF (**A**) or THF/water (**B**).

Without any annealing, a fingerprint pattern was observed for the thin film prepared from pure THF solution. However, the morphology changes to more spherical aggregates in the presence of water, which is in agreement with the previous TEM observations. It should be noted here that the sizes obtained from the AFM image (45 nm from THF/water film and 68 nm from pure THF film) were substantially smaller than those obtained from TEM and DLS measurements. This is primarily due to the change in solubility of PGMA-*g*-(PMMA-*b*-POEGMA) at higher concentration in the solvent mixture [29]. In addition, it has been reported that the concentration of the polymer can influence the size of the particles, so that increasing the viscosity of the polymerization medium decreases the particle size [41,42]. The surface properties of the block copolymer brush in the form of thin film were studied by water contact-angle measurements (Figure 10). As expected, the contact angle of the film made from THF/water system is significantly lower (45° ± 2°) than that made from pure THF (78° ± 2°). The presence of water is suspected to drive the hydrophilic parts POEGMA and possibly P(GMA-N_3_) at the interface, and thus persist in that configuration in the solid film, in agreement with the AFM observations.

## 3. Materials and Methods

### 3.1. Materials

Methyl methacrylate, glycidyl methacrylate, oligo(ethylene glycol) methyl ether methacrylate (OEGMA), 4-cyano-4(phenylcarbonothioylthio) pentanoic acid (CPADB) (>97%), azobisisobutyronitrile (AIBN), propargyl alcohol, sodium azide, *N*,*N’*-dicyclohexylcarbodiimide, 4-dimethylaminopyridine, copper(I)bromide, *N*,*N*,*N*′,*N*′′,*N*′′-pentamethyldiethylenetriamine (PMDETA), ammonium chloride, ammonia solution (25%), toluene, methanol, methylene chloride, diethyl ether, tetrahydrofuran, *N*,*N-*dimethylformamide, dimethyl sulfoxide, choloroform, and neutral alumina were purchased from Sigma-Aldrich and were used as received. NMR solvents CDCl_3_ and DMSO*-d_6_* were purchased from Eurisotop, Saint Aubin, France.

### 3.2. Characterization

^1^H-NMR Spectroscopy. ^1^H-NMR spectra were acquired in either CDCl_3_ or DMSO*-d*_6_ using a Bruker 300 MHz spectrometer (Billerica, MA, USA)**.**

Size Exclusion Chromatography (SEC). The SEC instrument of Viscotek (TDA 305, Malvern instruments, Worcestershire, UK) having a triple detector array was used to determine the average molecular weight of block copolymer and block copolymer brushes. The Viscotek SEC apparatus was equipped with two mixed-columns with a common particle size of 5 µm using THF as an eluent with flow rate of 1.0 mL/min. The number average molecular weight (*M_n_*) and dispersity index (*Ð*) were calculated thanks to universal calibration (RI and viscosimetry detectors) for homopolymers and measured with the multidetector systems for copolymers (RI, viscosimetry, light scattering).

Dynamic Light Scattering (DLS). DLS studies were used to measure the particle of the block copolymer brush using a Litesizer^TM^ 500 Zetasizer Nanoseries instrument from Anton Paar (Graz, Austria) equipped with a 40 mW He-Ne laser operating at 658 nm. The sample was analyzed in quartz cuvettes, and the temperature was set at 25 °C.

Transmission Electron Microscopy (TEM). TEM studies of the block copolymer brush were performed using a JEOL 1200 EXII instrument (JEOL, Croissy-sur-Seine, France) operating at 120 kV equipped with a numerical camera. About 5.0 μL of diluted block copolymer brush solution was placed on a top of the carbon-coated copper grid, which was then stained with 99.98% aqueous ammonium molybdate solution, and dried at room temperature.

Scanning Electron Microscopy (SEM). SEM analyses of the block copolymer brush were analyzed using a Hitachi S-4500 instrument (Tokyo, Japan) having spatial resolution of 1.50 nm at 15 kV energy. The samples were dried at room temperature and coated with an ultrathin layer of electrically conducting platinum deposited by evaporation under vacuum.

Atomic Force Microscopy (AFM). AFM images of the block copolymer brush were performed with an AFM Digital D 3100 (Digital Instruments, Bresso, Italy) in tapping and phase modes. Visible reflectance spectroscopy was performed using a reflectance fiber optic probe FCR-7IR200-2 from Avantes, a tungsten halogen light source (150 W), and an AvaSpec-2048TEC spectrometer (Avantes, Apeldoorn, The Netherlands).

Water Contact Angle (WCA). WCA was measured (GBX-Digidrop, Romans, France) by placing a water droplet of 3 μL onto the surface. The contact angle was measured using computerized image analysis.

### 3.3. Synthesis of POEGMA-b-PMMA-Alk, P(GMA-N_3_), and Block Copolymer Brush

#### 3.3.1. Synthesis of Propargyl Modified RAFT Agent (CPADB-Alk)

To a solution of 4-cyanoethyl thiocarbonyl pentanoic acid (0.5g, 0.00178 mol) and propargyl alcohol (0.1 g, 0.00179mol) in 10 mL of dichloromethane, DCC (0.74 g, 0.0035 mol) and DMAP (0.1 mol eq.) in 5 mL of dichloromethane were added drop-wise over a period of 15 min at 25 °C. The resulting mixture was stirred at this temperature for 12 h. The by-product dicyclohexylurea (DCU) was filtered off through the buchner funnel. The clear filtrate was concentrated to dryness under reduced pressure to give a pink crude solid. The solid was dissolved in 2 mL of dichloromethane and still contains some of the insoluble impurity, which was filtered off. To the clear filtrate, 5% n-Hexane was added and stored in a freezer overnight before filtration. The filtrate was concentrated to give the desired product as a pink oil. (80% yield, >95% purity by ^1^H-NMR). ^1^H-NMR (CDCl_3_, δ in ppm): 1.86 (s, CCNC*H_3_*), 2.45 (t,C≡C*H*), 2.5–2.8 (m, -C*H_2_*C*H_2_*-), 4.7 (d,-C(O)-O-C*H_2_*), 7.2–7.8(m, 5*H*). ^13^C-NMR (CDCl_3_, δ in ppm): 24.26 (C-CH_3_), 29.92 (CH_2_-C(=O)), 33.23 (C-CH_2_-CH_2_), 45.69 (C-CN), 63.69 (O-CH_2_-C), 75.86 ((C≡CH), 77.56 (C≡CH), 118.42 (CN), 126.62 (C aro.), 128.48 (C aro.), 132.93 (C aro.), 144.54 (C aro.), 170.82 (C(=O)), 222.19 (C(=S)-S). ν_max_/cm^−1^ 3280, 2119, 1715, 1443, 1391, 1262, 1171, 1102, 1079, 1048, 1003, 846, 804, 763, 693. *m/z* (ESI) 340.1 [M − Na]^+^ C_16_H_15_NO_2_S_2_ calcd 340.04).

#### 3.3.2. Synthesis of Alkyne Terminated Poly(methyl methacrylate) Macro-Chain Transfer Agent (PMMA-Alk)

Synthesis of alkyne terminated PMAA macro-CTA was performed as follows: methyl methacrylate (MMA; 0.36 g; 3.65 mmol), CPADB-Alk (22 mg; 0.073 mmol), and azobisisobutyronitrile (AIBN) (3 mg; 0.0183 mmol) were allowed to dissolved in toluene (6.0 g). The mixture was thoroughly purged with oxygen-free nitrogen and then immersed into an oil bath at 70 °C for 4 h. The polymerization was stopped by suddenly cooling the reaction mixture to 10 °C. The reaction mixture was diluted with methylene chloride before precipitation into 10-fold excess of methanol. The dissolution–precipitation procedure was repeated three times. The obtained solid was then dried at 25 °C under reduced pressure for 24 h (m = 0.24 g, 72% conversion determined by ^1^H-NMR spectroscopy in CDCl_3_. M_n,SEC_ = 1800 g·mol^−1^, Ð = 1.09)**.**

#### 3.3.3. Synthesis of Poly(oligo(ethylene glycol) methyl ether methacrylate)-*b*-Poly(methyl methacrylate) (POEGMA-*b*-PMMA-Alk) Block Copolymer

PMMA-Alk (M_n,SEC_ = 1800 g·mol^−1^, Ð = 1.09, 0.175g, 0.0975 mmol), OEGMA (2.9 g, 9.75 mmol), and AIBN (4 mg, 0.0243 mmol) were dissolved in 9 g of toluene in a flask. The solution was degassed by nitrogen and then immersed in a preheated oil bath at 70 °C for 30 min. The reaction was ended by cooling the flask in ice water. The polymer solution was poured into a large excess of *n*-hexane mixture to precipitate POEGMA-*b*-PMMA-Alk block copolymer. The precipitation procedure was repeated twice. The block copolymer was dried under vacuum to a constant weight and characterized by ^1^H NMR spectroscopy and SEC (m = 0.61 g, 22% conversion as determined by ^1^H-NMR spectroscopy in CDCl_3_. M_n,SEC_ = 3100 g·mol^−1^, Ð = 1.13)**.**

#### 3.3.4. Synthesis of Poly(glycidyl methacrylate) (PGMA)

Synthesis of PGMA was performed as follows: glycidyl methacrylate (GMA, 1.039 g, 7.3 mmol), 4-cyano-4-(phenylcarbonothioylthio)pentanoic acid (20 mg; 0.073 mmol), and AIBN (3 mg; 0.0183 mmol) were dissolved in THF (12.0 g). The mixture was thoroughly degassed with pure nitrogen and then immersed into an oil bath at 65 °C for 1 h. After 1 h, the polymerization was stopped by suddenly cooling the reaction mixture to approximately 10 °C. The reaction mixture was then diluted with methylene chloride before precipitation into 10-fold excess diethyl ether. The collected solid was dissolved in methylene chloride and precipitated again. The dissolution-precipitation procedure was repeated three times. The pale pink solid after precipitation was dried at 25 °C under vacuum for 5 h (m = 0.42g, 52% conversion as analysed by ^1^H-NMR spectroscopy in DMSO-*d*_6_. M_n,SEC_ = 3300 g·mol^−1^, Ð = 1.12).

#### 3.3.5. Synthesis of Poly(glycidyl methacrylate-azide) (P(GMA-N_3_))

P(GMA-N_3_) was synthesized through the ring-opening of GMA units. A mixture of PGMA (0.259 g, M_n,SEC_ = 3300 g·mol^−1^, Ð = 1.12), NaN_3_ (0.284 g, 4.37 mmol), NH_4_Cl (0.234 g, 4.37 mmol), and DMF (5 mL) was stirred in a round bottom flask at 50 °C for 24 h. The reaction mixture was filtered to remove the inorganic salt and then precipitated in excess water, filtered, and washed with deionized water several times. The product was dried in a vacuum oven at 50 °C for 24 h (m = 0.35 g, M_n,SEC_ = 5200 g·mol^−1^, Ð = 1.15).

#### 3.3.6. Synthesis of PGMA-*g*-(PMMA-*b*-POEGMA) Block Copolymer Brush

The click reactions of P(GMA-N_3_) (M_n,SEC_ = 5200 g·mol^−1^, Ð = 1.13) and POEGMA-*b*-PMMA-Alk block copolymer (M_n,SEC_ = 3100 g·mol^−1^, Ð = 1.13) were conducted as follows: P(GMA-N_3_) (0.1 g, 0.019 mmol, 1.0 mol eq.), POEGMA-*b*-PMMA-Alk (0.12 g, 0.038 mmol, 2.0 mol eq.), and PMDETA (0.016 g, 5.0 mol eq.) were dissolved in 10 mL of THF and purged with N_2_ bubbling for 30 min. CuBr (0.013 g, 5.0 mol eq.) was introduced under N_2_ to the solution, which quickly turned green. After stirring for 20 h at 35 °C, the solution was passed through an aluminium oxide column to remove the copper impurity and the solvent was evaporated under reduced pressure. The crude product was dissolved in THF and precipitated in diethyl ether. After filtration, the collected solid was dried under vacuum at 25 °C. It was then dissolved in dichloromethane, washed with ammonia solution, and the dichloromethane phase was concentrated under reduced pressure. The polymer was further purified by dialysis against THF using dialysis tubing with a molecular weight cut-off of 12,000 Da for one day and then freeze dried. The obtained viscous liquid was then dissolved in THF and stirred in diethyl ether, filtered, and vacuum-dried (m = 0.14 g, M_n,SEC_ = 86,000 g·mol^−1^, Ð = 1.32).

### 3.4. Thin Film Preparation

PGMA-*g*-(PMMA-*b*-POEGMA) block copolymer brush was dissolved in THF or THF/water mixture and filtered using a disposable syringe having a pore size of 0.45 μm, producing a 15.0 wt% solution. The viscous solutions were spin-coated using an SPS Spin 150 Spin coater at 1000 revolutions per minute (rpm) for 120 s with a speed ramp of 100 rpm s^−1^ onto precleaned silicon substrates and dried under nitrogen atmosphere for 4 h.

## 4. Conclusions

Well-defined PGMA-*g*-(PMMA-*b*-POEGMA) block copolymer brush was synthesized using click chemistry and reversible addition fragmentation chain transfer (RAFT) polymerization and characterized by ^1^H-NMR and SEC. Upon treatment with selective solvents, the block copolymer brushes exhibited different morphologies, which were monitored using DLS and TEM analyses. Thin film of block copolymer brush was also prepared using the spin coating technique. A uniform morphology and a smooth surface were observed by SEM analysis. AFM analysis of the treated block copolymer brush indicated that spherical morphology aggregates were formed when water was used as a selective solvent. Contact angle measurements further confirm the surface organization of the block copolymer brush with the hydrophilic blocks oriented towards the surface. Thanks to the aggregated nature of the block copolymer brush, hydrophilic percolating pores are expected and a further study will explore the use of block copolymer brush films as a filtration membrane for aqueous solutions.

## Figures and Tables

**Table 1:** Structural characteristics of all brush block copolymers. POEGMA-*b*-PMMA, poly(oligo(ethylene glycol) methyl ether methacrylate)-*b*-poly(methyl methacrylate); PGMA, poly(glycidyl methacrylate).

Sample Code	PMMA-Alky		PGMA		P(GMA-N_3_)		POEGMA-*b*-PMMA-Alk		Block Copolymer Brush	
	M_n_ (g/mol)	Ð	M_n_ (g/mol)	Ð	M_n_ (g/mol)	Ð	M_n_ (g/mol)	Ð	M_n_ (g/mol)	Ð
1: PGMA_23_-*g*-(PMMA_18_-*b*-POEGMA_4_)	1800	1.09	3300	1.12	5200	1.13	3100	1.13	86,000	1.32
2: PGMA_31_-*g*-(PMMA_33_-*b*-POEGMA_4_)	3300	1.15	4400	1.17	6500	1.22	4600	1.20	148,000	1.27
3: PGMA_10_-*g*-(PMMA_33_-*b*-POEGMA_4_)	3300	1.15	1400	1.15	2150	1.19	4600	1.20	47,800	1.26
4: PGMA_23_-*g*-(PMMA_33_-*b*-POEGMA_9_)	3300	1.15	3300	1.12	5200	1.13	6000	1.21	153,000	1.33

## Supplementary Materials

The following are available online. Figure S1. Disappearance of the RAFT end group signal as evidenced by ^1^H-NMR.
