# Supplementary material for: Synthesis of Block Copolymer Brush by RAFT and Click Chemistry and Its Self-Assembly as a Thin Film
Source: Molecules. 2020 Oct 17;25(20):4774. doi: 10.3390/molecules25204774 (PMC7587578; doi:10.3390/molecules25204774)

Supporting Information

Synthesis of block copolymer brush by RAFT and click chemistry and its self-assembly as a thin film

Hajeeth Thankappan, Mona Semsarilar, Suming Li, Antoine Venault, Yung Chang, Denis Bouyer and Damien Quemener *

Figure S1. Disappearance of the RAFT end group signal as evidenced by H^1 NMR

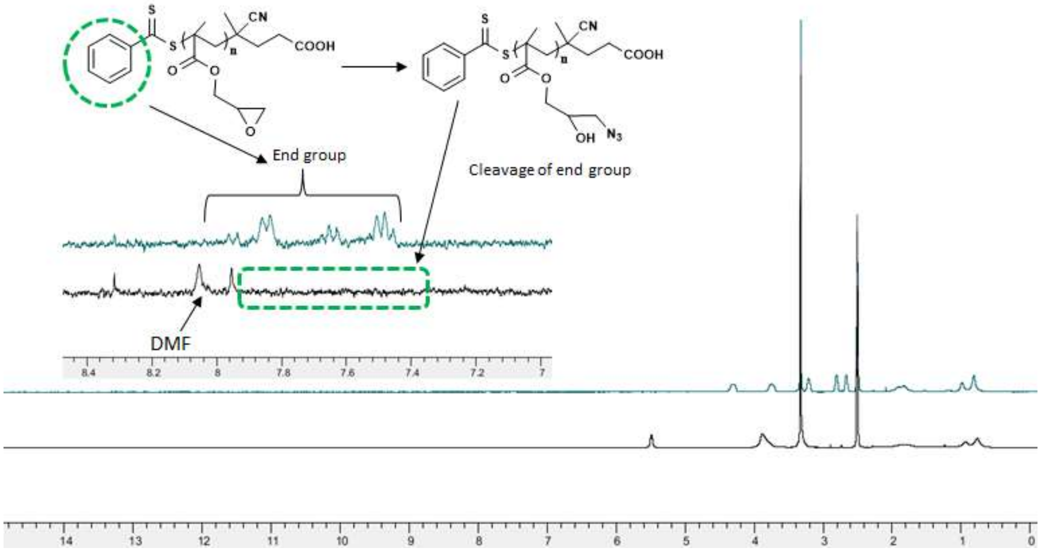

Supplement: Supplementary file 1 [file molecules-25-04774-s001.pdf]
